# Angiogenic Potential of 3-Nitro-4-Hydroxy Benzene Arsonic Acid (Roxarsone)

**DOI:** 10.1289/ehp.10885

**Published:** 2008-01-17

**Authors:** Partha Basu, Richik N. Ghosh, Linnette E. Grove, Linda Klei, Aaron Barchowsky

**Affiliations:** 1 Department of Chemistry and Biochemistry, Duquesne University, Pittsburgh, Pennsylvania, USA; 2 Thermo Fisher Scientific, Rockford, Illinois, USA; 3 Thermo Fisher Scientific, Pittsburgh, Pennsylvania, USA; 4 Department of Environmental and Occupational Health, University of Pittsburgh, Pittsburgh, Pennsylvania, USA

**Keywords:** angiogenesis, animal feed, arsenic, gene expression, nitric oxide, roxarsone, tube formation

## Abstract

**Background:**

Roxarsone (3-nitro-4-hydroxy benzene arsonic acid) is an arsenic compound widely used in the poultry industry as a feed additive to prevent coccidiosis, stimulate growth, and to improve tissue pigmentation. Little is known about the potential human health effects from roxarsone released into the environment from chicken waste or from residual compound in chicken products.

**Objective:**

The growth potentiation and enhanced tissue pigmentation suggest that low levels of roxarsone exposure may have an angiogenic potential similar to that of inorganic arsenite (As^III^). The goal of this investigation was to test the hypothesis described above using cultured human aortic and lung microvascular endothelial cells in high-content imaging tube-forming assays and begin developing a molecular level understanding of the process.

**Methods:**

We used a three-dimensional Matrigel assay for probing angiogenesis in cultured human endothelial cells, and a polymerase chain reaction (PCR) array to probe the gene changes as a function of roxarsone or As^III^ treatment. In addition, we used Western blot analysis for changes in protein concentration and activation.

**Results:**

Roxarsone was found to exhibit a higher angiogenic index than As^III^ at lower concentrations. Increased endothelial nitric oxide synthase (eNOS) activity was observed for roxarsone but not for As^III^-induced angiogenesis. However, As^III^ caused more rapid and pronounced phosphorylation of eNOS. Quantitative PCR array on select genes revealed that the two compounds have different and often opposite effects on angiogenic gene expression.

**Conclusions:**

The results demonstrate that roxarsone and As^III^ promote angiogenic phenotype in human endothelial cells through distinctly different signaling mechanisms.

Arsenic contamination of drinking water is a worldwide public health concern. The extent of the concern in some countries has reached a point such that some are calling this humanity’s largest mass poisoning (Bhattacharjee 2007). Even low levels of exposure to arsenic have been linked to increased cardiovascular disease and hypertension (Chen et al. 2007; Navas Acien et al. 2005). Although natural contamination of drinking water with inorganic arsenic represents the largest arsenical hazard to human health, environmental exposure to commercial organoarsenicals is a growing concern (Sapkota et al. 2007). Organoarsenicals such as roxarsone are widely used by the poultry industry (Arai et al. 2003; Brown et al. 2004; Chapman et al. 2002), with approximately 2.2 million pounds of roxarsone being fed to broiler chickens raised in the United States per year (Garbarino et al. 2003). The majority of roxarsone is excreted unchanged from chickens (Morrison 1969), while the remainder increases the total arsenic present in chicken tissue (Lasky et al. 2004). This residual roxarsone could amount to ingestion of 1.38–5.24 mg/day of arsenic at mean levels of chicken consumption (60 g/person/day) (Wallinga 2006). The excreted roxarsone may pose an environmental hazard, as microbes, including those residing in the gut microflora, can release inorganic arsenite (As^III^) from roxarsone (Stolz et al. 2007). The human health impacts of roxarsone have not been well studied, and the mechanisms for its biological effects in mammalian tissues are unknown. It has been suggested, however, that all of these biological effects require metabolism to inorganic As^III^ (O’Connor et al. 2005).

In a variety of *in vivo* and *in vitro* models, nanomolar or low micromolar concentrations of arsenic (As^III^) stimulate angiogenesis and vascular remodeling that may promote vascular diseases and tumorigenesis (Kamat et al. 2005; Liu et al. 2006; Soucy et al. 2003, 2005). In addition to enhancing tumor growth, increased angiogenesis would contribute to overall growth potential and increased tissue pigmentation. These are the attributes of roxarsone that contribute to its widespread use; however, the cellular effects of roxarsone to mammalian cells are not known. Further, it is unclear whether the vascular effects of roxarsone are dependent on its metabolism to inorganic arsenic. Herein we report the angiogenic potential of roxarsone and compare it with that of inorganic arsenite (As^III^). In addition, we report different modes of action of these two compounds in promoting angiogenesis.

## Materials and Methods

### Culture of endothelial cells

Human aortic endothelial cells (HAEC) and lung microvascular endothelial cells (HMVEC) (Clonetics; Lonza, Walkersville, MD, USA) were cultured at 5% CO_2_ in complete MCDB 131 medium (Invitrogen, Carlsbad, CA, USA) supplemented with 5% fetal calf serum (Hyclone; Thermo Fisher Scientific, Pittsburgh, PA, USA), 1% pen/strep, 1% hydrocortisone, 2 mM l-glu-tamine, and 10 ng/mL epidermal growth factor (Sigma-Aldrich Chemical Co., St. Louis, MO, USA). Under these conditions up to 10 μM roxarsone was not cytotoxic, as determined by dye exclusion assays, whereas As^III^ was toxic at 10 μM but not at 5 μM (Barchowsky et al. 1999a). Cells were used at passages 6–7 in three-dimensional Matrigel matrix cultures to probe the angiogenic potential (tube formation) of roxarsone and As^III^.

### Three-dimensional angiogenic tube-formation assay

Concentration-responsive effects of roxarsone and As^III^ on the angiogenic potential of HAEC and HMVEC were compared in quantitative high-content cellular imaging tube-formation assays in Matrigel (BD Biosciences, San Jose, CA, USA). Cells were incubated for 24 hr in reduced serum and growth factor MCDB 131 (1:5 dilution of complete MCDB 131 with nonsupplemented MCDB 131). The cells were then released from the culture dish with trypsin, diluted in MCDB 131 with or without inhibitors, and 6,000–10,000 cells were plated onto 35-mL Matrigel cushions in 96-well plates. Sodium arsenite (As^III^)) or roxarsone was then added from 1,000× stock solutions. As positive controls for angiogenic tube formation, either vascular endothelial growth factor (1 ng/mL) or a cocktail of growth factors (vascular endothelial growth factor, 10 ng/mL; fibro-blast growth factor, 10 ng/mL; erythropoietin, 2 U/mL; and interleukin-6, 10 ng/mL) were added to the cultures. After 16 hr, the medium was removed and the gels were air dried. Rhodamine-labeled phalloidin and 4′-6-diamidino-2-phenylindole (DAPI; (Sigma-Aldrich) were added to stain F-actin and nuclei, respectively. Images of fluorescently labeled cells were collected with a Thermo Scientific Cellomics ArrayScan HCS Reader (Thermo Fisher Scientific, Pittsburgh, PA, USA) and analyzed by an automated algorithm that identified the tubes formed by the association and clustering of the endothelial cells. This algorithm provided quantitative measurements of tube properties such as the number of tubes, tube lengths, tube areas, number of nodal branch points, and the angiogenic index—defined as the percentage of image area covered by tubes multiplied by 10 (Ghosh et al. 2007; Grove and Ghosh 2006). Each experiment was repeated 3 times, and each treatment was probed in at least six wells. Differences between treatments were analyzed by one- and two-way analysis of variance (ANOVA) and Dunnett or Bonferroni post hoc tests for significance using Graphpad Prism v4.0 software (Graphpad Software, San Diego, CA, USA).

### SuperArray Angiogenesis RT2 Profiler PCR array

The effects of 24-hr exposures to As^III^ and roxarsone on HMVEC mRNA levels for 84 angiogenic and 5 constitutive genes were measured using the SuperArray quantitative polymerase chain reaction (PCR) assay (SuperArray Bioscience Corp., Frederick, MD, USA). Total RNA was extracted from approximately 500,000 cells using QIAGEN RNeasy (QIAGEN, Chatsworth, TN, USA) mini kits with inclusion of a DNase treatment step. First-strand cDNA synthesis and quantitative real-time PCR with SYBR green master mix was performed in an Opticon 2 DNA engine (BioRad Corp., Hercules, CA, USA) according to the manufacturer’s instructions.

### Western analysis

Western blot analysis was performed on proteins isolated from HAEC exposed to As^III^ or roxarsone for 0.5, 1, or 4 hr. The proteins were separated by sodium dodecyl sulfate–polyacrylamide gel electrophoresis and transferred to polyvinylidene membranes, as previously described (Barchowsky et al. 1999b). Blots were probed with primary antibody to either total or ser1177 phosphorylated eNOS (Cell Signaling Technologies, Danvers, MA, USA) and reacted bands were detected by horseradish peroxidase–conjugated secondary antibodies and enhanced chemiluminescence substrates (PerkinElmer, Boston, MA, USA). Density of bands on film exposed to blots was determined using Scion Image software (Scion Corp., Frederick, MD, USA).

## Results and Discussion

**Figure 1:**
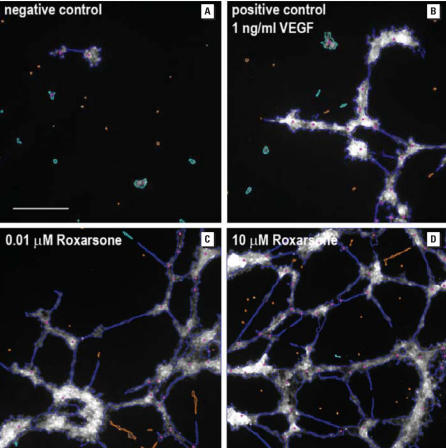
Images of fluorescent-labeled HAEC (10,000 cells/well) treated with different concentrations of roxarsone. HAEC were grown on a thin layer of Matrigel in a 96-well plate, treated for 16 hr, then fixed and fluorescently labeled with rhodamine–phalloidin (λ*_ex_* ~ 542 nm, λ*_em_* ~ 565 nm) to delineate the cell and tube structures. Images were acquired on a Thermo Scientific Cellomics ArrayScan HCS Reader using a 5× microscope objective (lengths of images shown are 1,400 μm, and scale bar in the negative control panel is 350 μm), and analyzed by an automated algorithm for quantifying tube morphology. Grayscale fluorescence images are shown with colored overlays from the algorithm identifying the different entities measured: connected tubes are outlined in blue, unconnected objects are in aqua, nodes where the tube branches are marked by pink dots, and objects rejected from the quantitative analysis are in orange.

**Figure 2:**
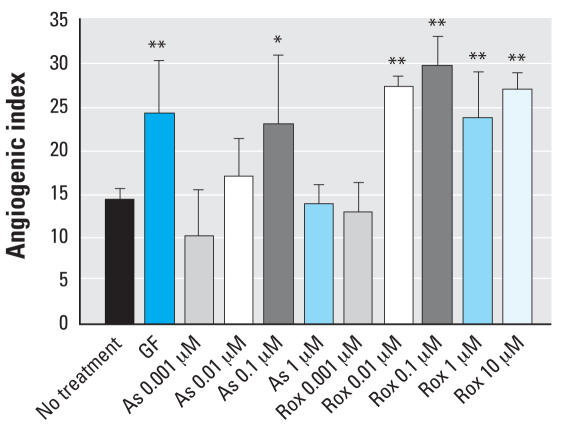
Angiogenic index obtained directly from the image processing of the tube formation assay on HAEC. “No treatment” refers to the negative control, which had no treatment, and the cells were grown on serum-free basal media; GF refers to a mixture of growth factors used as a positive control (VEGF, 10 ng/mL; fibroblast growth factor, 10 ng/mL; erythropoietin, 2 U/mL; and interleukin-6, 10 ng/mL). As and Rox refer to As^III^ and roxarsone, respectively. In each case, data were collected from six wells, and five different fields were imaged for each well. The angiogenic index ± SD is plotted for each condition. Error bars indicate SDs. *p < 0.05 and **p < 0.01 determined from one-way ANOVA analysis, followed by the Dunett post-test.

Concentration-responsive effects of roxarsone and As^III^ on the angiogenic potential of HAEC and HMVEC were compared in quantitative high-content cellular imaging tube-formation assays in Matrigel (Figure 1). Images of fluorescently labeled cells were analyzed by an algorithm that identified the angiogenic tubes formed by the association and clustering of the endothelial cells and quantitatively measured the properties of the identified tubes such as the number of tubes, tube lengths, tube areas, number of nodal branch points, and the angiogenic index. The angiogenic index is defined as the percentage of image area covered by tubes multiplied by 10 (Ghosh et al. 2007; Grove and Ghosh 2006). Both compounds were as effective as vascular endothelial cell growth factor (VEGF) in increasing the angiogenic index above the value for untreated control cells in both cell types (Figures 1 and 2). Both cell types exhibited strong angiogenic responses with the arsenical treatments; however, the HMVEC showed a higher basal level of angiogenesis (Table 1). Because of their lower basal angiogenic activity, HAEC were used for additional mechanistic studies.

The angiogenic threshold for roxarsone was between 0.001 and 0.01 μM, an order of magnitude lower than the threshold for As^III^ (Figures 1 and 2). As we have previously shown in *in vivo* chicken allantoic membrane angiogenesis assays (Soucy et al. 2003), above 1 μM As^III^ becomes toxic to tube formation and inhibits angiogenesis (Figure 2). In contrast, even 10 μM roxarsone showed no decrease in the angiogenic index (Figures 1 and 2) or signs of toxicity in the endothelial cells (data not shown). The fact that the efficacy for tube formation was similar, but the toxic potential differs, suggested that the two arsenicals differ in their mechanisms of action.

**Figure 3:**
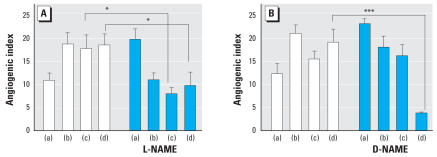
Effects of L-NAME (*A* ) and D-NAME (*B* ) on angiogenic index. HAEC were treated as follows: (a) no treatment, (b) a mixture of growth factors as a positive control, (c) roxarsone (0.1 μM), and (d) As^III^ (0.1 μM). Open bars represent cells with no NAME; blue bars represent cells treated with different isomers of NAME (50 μM). In each case, 12 wells were investigated, and each well was imaged at five different fields. Angiogenic index ± SD is plotted in each case. *p < 0.05 and ***p < 0.001 determined from two-way ANOVA analysis, followed by the Bonferroni post-test.

Nitric oxide (NO) production is required for certain stimuli to promote angiogenesis (Yu et al. 2005), but As^III^ is known to promote reactive oxygen that limits available NO release in endothelial cells (Barchowsky et al. 1999a, Bunderson et al. 2006). The data in Figure 3 demonstrate that roxarsone requires NO generation to increase angiogenesis. Its effects were blocked by the eNOS inhibitor l-NG-nitroarginine methylester (L-NAME), but not its inactive enantiomer d -NG-nitroarginine methylester (D-NAME). In contrast, both NAME enantiomers prevented As^III^ from increasing the angiogenic index. This would suggest either that As^III^ inhibition by NAME was a nonstereospecific chemical effect or that D-NAME interacted with As^III^ in a unique manner relative to roxarsone. Thus, roxarsone and inorganic As^III^ appeared to have differential effects on signaling for NO-mediated angiogenesis.

**Figure 4:**
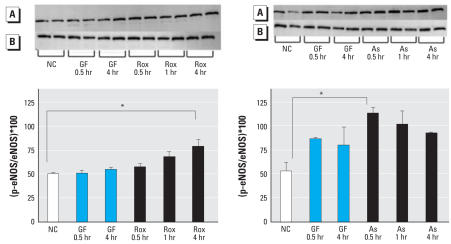
Western blot showing phosphorylated eNOS (p-eNOS) (*A*), and total eNOS (*B*) in HAEC. Proteins were loaded equally after protein determination, which was confirmed by probing beta-actin (data not shown). Conditions: NC, no additional treatment; GF, a mixture of grown factors as positive control as described in Figure 2; Rox, 1 μM roxarsone; As, 1 μm As^III^. All lanes on the gels were generated using proteins from separate cell cultures. Two replicates were done for each condition and were also used for statistical analyses. Individual bands were analyzed using Scion Image program, and the percent eNOS phosphorylated with respect to total is plotted for each condition (mean ± SD). *p < 0.05 and ***p < 0.001 determined from two-way ANOVA analysis, followed by the Dunnett post-test.

To investigate a possible mechanism for increased eNOS activity after exposure to arsenicals, we exposed HAEC to either roxarsone or As^III^ for 0.5–4 hr, then probed for levels of serine phosphorylated eNOS relative to total eNOS. As shown in Figure 4, both arsenicals caused time-dependent increases in Ser1177 phosphorylation of eNOS. However, roxarsone caused a progressive increase over 4 hr, whereas As^III^ caused a rapid stimulation of phosphorylation that was declining by 4 hr. These data indicate that the arsenicals do not share similar common upstream signaling actions that enhance the activity of AKT, a serine/threonine protein kinase, which commonly accounts for serine phosphorylation (Dimmeler et al. 1999; Fulton et al. 1999). In addition, the time course for the effects of As^III^ is consistent with its known ability to stimulate vascular NADPH oxidase to generate superoxide that quenches NO to form peroxynitrite (Bunderson et al. 2002; Lynn et al. 2000; Smith et al. 2001). These oxidants often mediate inflammatory angiogenesis (Ushio-Fukai and Alexander 2004) and thus may account for the mechanism of action for As^III^.

Analysis of the effects of the two arsenicals on angiogenic gene activity further indicated that roxarsone and As^III^ signal through distinct but overlapping mechanisms. Superarray quantitative PCR arrays of total RNA extracts from HMVEC treated with either arsenical for 24 hr demonstrated that the arsenicals differentially affect expression of a limited set of 9 of 84 inducible genes measured. As^III^ induced more genes than it inhibited (Table 2). At 0.1 μM, roxarsone inhibited more genes than it induced (Table 2). Several genes were affected in the same direction, but not to the same degree by the two agents. Both arsenicals decreased angiogenic repressive interferon 1_α_ (Strieter et al. 2005) and interferon-inducible CXCL9 transcripts. It was interesting that generally the higher roxarsone concentration (1.0 μM) had either no effect on gene expression or the opposite effect of the low concentration. The exception was the concentration-dependent induction of hepatocyte growth factor (HGF) mRNA levels by both roxarsone concentrations. As^III^ also strongly induced HGF transcripts. It is difficult to reconcile the differential effects of As^III^ and the two concentrations of roxarsone on gene expression with their concentration-dependent effects on angiogenic potential. It is possible that induction of a dominant factor, such as HGF, accounted for the observed increases in tube formation. However, the analysis was limited by the small number of gene changes measured and a lack of information regarding protein changes that may account for increased angiogenic index. Although beyond the scope of the current studies, additional mechanistic experiments would delineate the linkage between any gene or protein change and increased angiogenic index after arsenical exposure. Nonetheless, these data demonstrate that roxarsone and inorganic As^III^ signal through different mechanisms to affect induction of genes that regulate angiogenesis.

## Conclusion

Our results in human endothelial cell cultures demonstrate that roxarsone was a more potent inducer of angiogenesis than inorganic As^III^, and that roxarsone and As^III^ signal through separate pathways to promote angiogenesis. The arsenicals had different time courses for phosphorylating eNOS; only the effects of roxarsone were blocked specifically by eNOS inhibition, and the two arsenicals differentially affected angiogenic gene induction. In addition to these differential effects, the fact that roxarsone is more potent for tube formation and less cytotoxic supports the conclusion that metabolism to inorganic As^III^ cannot account for its vascular effects. More importantly, this was the first demonstration of functional vascular effects of roxarsone that may implicate a disease risk. Future studies are needed to demonstrate the mechanisms that enable differential signaling by the two arsenicals and the impact of this signaling in human endothelial cells on vascular disease promoted by their environmental exposures.

## Figures and Tables

**Table 1:** Angiogenic index of the response to arsenicals by different endothelial cells.

	HAEC		HMVEC	
Treatment	Mean ± SD	Negative control (%)	Mean ± SD	Negative control (%)
No treatment (negative control)	15 ± 3	100	95 ± 62	100
Growth factor (positive control)	35 ± 28	239	125 ± 75	132
As^III^ (0.1 μM)	32 ± 29	223	146 ± 81	155
Roxarsone (0.1 μM)	28 ± 9	191	198 ± 95	210

**Table 2:** SuperArray Angiogenesis RT^2^ Profiler PCR array for angiogenic genes.

	Fold control		
Genes	0.1 μM roxarsone	1.0 μM roxarsone	1.0 μM As^III^
Proangiogenic genes			
Angiopoietin-1	0.46 ± 0.25*	0.77 ± 0.25	0.67 ± 0.14
CXCL3	0.95 ± 0.25	1.38 ± 0.52	1.74 ± 0.10*
Hepatocyte growth factor	1.99 ± 0.63*	2.70 ± 0.38**	4.20 ± 0.38***
Insulin-like growth factor 1	0.84 ± 0.54	0.90 ± 0.37	2.47 ± 0.63**
Leukocyte cell–derived chemotaxin 1	0.46 ± 0.23*	0.96 ± 0.20	0.55 ± 0.09*
Leptin	0.21 ± 0.08**	0.98 ± 0.43	0.57 ± 0.29
Plasminogen	0.31 ± 0.02**	0.84 ± 0.18	0.63 ± 0.30
Anti-angiogenic genes			
Interferon 1_±_	0.34 ± 0.09*	1.14 ± 0.39	0.71 ± 0.32
CXCL9	0.17 ± 0.08**	1.03 ± 0.33	0.46 ± 0.13**

Approximately 500,000 HMVEC were incubated with roxarsone or As^III^ for 24 hr. Extracted total RNA was then isolated and mRNA levels for 84 inducible and 5 constitutive genes were measured, according to the manufacturer’s instructions. Data are presented as mean ± SD fold over untreated control for PCR products normalized to housekeeping gene expression.

* *p* < 0.05,

** *p* < 0.01, and

*** *p* < 0.001 (*n* = 6 separate RNA extracts).
